# Crystal structure of 1,1′,2,2′,4,4′-hexa­iso­propyl­magnesocene

**DOI:** 10.1107/S2056989022001189

**Published:** 2022-02-03

**Authors:** Nico Bachmann, Lisa Wirtz, Bernd Morgenstern, Carsten Müller, André Schäfer

**Affiliations:** a Saarland University, Faculty of Natural Sciences and Technology, Department of Chemistry, Campus Saarbrücken, 66123 Saarbrücken, Germany

**Keywords:** crystal structure, magnesocene, cyclo­penta­dienide

## Abstract

The title compound, ^3^Cp_2_Mg or [Mg(C_14_H_23_)_2_], was synthesized from the cor­res­ponding triiso­propyl­cyclo­penta­diene by treatment with *n*-butyl-*sec*-butyl­magnesium. The structural characterization by single-crystal X-ray diffraction revealed that the compound crystallizes in the triclinic space group *P*  with half a mol­ecule per asymmetric unit and a staggered arrangement of the cyclo­penta­dienide ligands.

## Abstract 2

The title compound was synthesized from the corresponding triiso­propyl­cyclo­penta­diene by treatment with *n*-butyl-*sec*-butyl­magnesium and crystallizes in the triclinic space group *P* 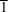 with half a mol­ecule per asymmetric unit and a staggered arrangement of the cyclo­penta­dienide ligands.

## Chemical context

Magnesocene (Cp_2_Mg) was initially reported by Wilkinson and Fischer and co-workers in 1954, just a few years after the discovery of ferrocene (Wilkinson & Cotton, 1954 ▸; Fischer & Hafner, 1954 ▸). Although magnesocene exhibits distinctively different chemical properties, it is isostructural to ferrocene and marked the beginning of main-group metallocene chemistry. One of the key differences in reactivity between alkaline-earth metallocenes and ferrocenes is that the central atoms of the former exhibit Lewis acidic character. Therefore, many crystal structures of magnesocenes are actually of donor complexes, such as magnesocene mono- and bis­(tetra­hydro­furan) adduct, Cp_2_Mg**·**(thf) and Cp_2_Mg**·**(thf)_2_ (Lehmkuhl *et al.*, 1986 ▸; Jaenschke *et al.*, 2003 ▸; Kim *et al.*, 2007 ▸). Nevertheless, solvent-free crystal structures are also known, especially in case of highly substituted magnesocenes (Morley *et al.*, 1987 ▸; Gardiner *et al.*, 1991 ▸; Weber *et al.*, 2002 ▸; Vollet *et al.*, 2003 ▸; Deacon *et al.*, 2015 ▸; Müller *et al.*, 2021 ▸). Hanusa and coworkers had reported the synthesis of 1,1′,2,2′,4,4′-hexa­iso­propyl­magnesocene, ^3^Cp_2_Mg, -calcocene, ^3^Cp_2_Ca, -strontocene, ^3^Cp_2_Sr, and -barocene, ^3^Cp_2_Ba (the triiso­propyl­cyclo­penta­dienide ligand is commonly abbreviated as ‘^3^Cp’), *via* treatment of potassium 1,2,4-triiso­propyl­cyclo­penta­dienide, ^3^CpK, with the corresponding metal(II) bromide or iodide and described the magnesocene to be oily or waxy in composition (Burkey *et al.*, 1993 ▸, 1994 ▸). Thus, no crystal structure was obtained of the title compound. We found that the title compound may also be obtained through treatment of an isomeric mixture of triiso­propyl­cyclo­penta­diene with *n*-butyl-*sec*-butyl­magnesium in hexane. 
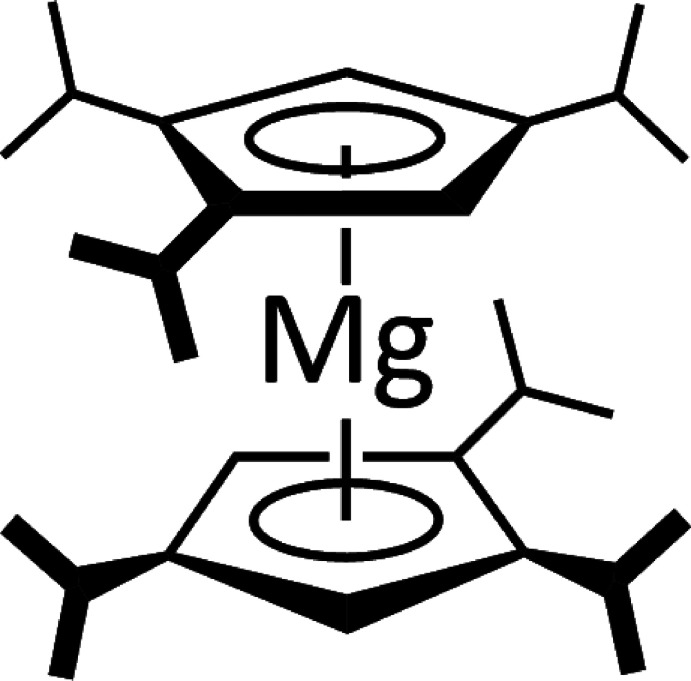


## Structural commentary

**Figure 1:**
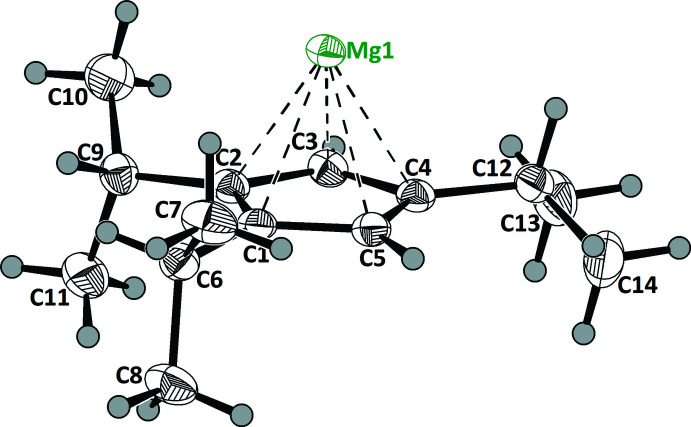
Asymmetric unit of the title compound (displacement ellipsoids are drawn at the 50% probability level).

**Figure 2:**
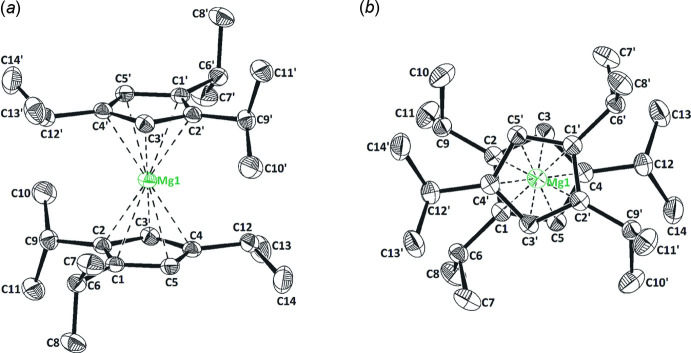
(*a*) Side view and (*b*) top view of the mol­ecular structure of the title compound in the crystal. Symmetry code: (’) 1 − *x*, 1 − *y*, 1 − *z*. Displacement ellipsoids are drawn at the 50% probability level; H atoms omitted for clarity.

The title compound crystallizes in the triclinic space group *P* 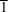 with half a mol­ecule per asymmetric unit, due to an inversion center at the magnesium atom position (Fig. 1 ▸), resulting in *C*_2*h*_ symmetry for the mol­ecule. Accordingly, the Cp rings adopt a staggered arrangement with the single isopropyl group at the C4 position facing the two isopropyl groups at the C1 and C2 positions and are perfectly coplanar to each other (Fig. 2 ▸). The C—C bond lengths within the Cp ring are almost equal [C1—C2: 1.4237 (18) Å; C2—C3: 1.4268 (17) Å; C3—C4: 1.4172 (19) Å; C4—C5: 1.4220 (18) Å; C5—C1: 1.4277 (18) Å] implying a high degree of 6π electron aromaticity, and the Mg⋯Cp^centroid^ distance is 1.9852 (1) Å, which is within the normal range [*e.g.*: Cp_2_Mg: 1.9897 (5) Å] for magnesocenes (Bünder & Weiss, 1975 ▸).

## Supra­molecular features

**Figure 3:**
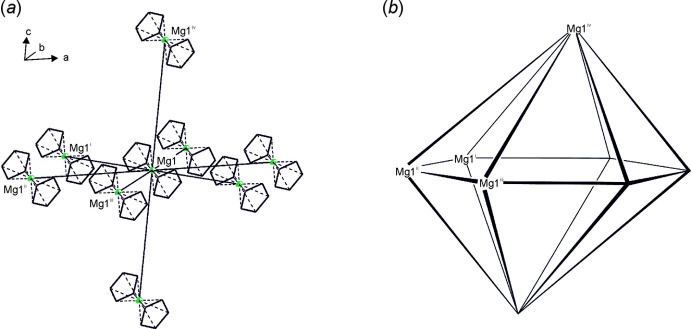
(*a*) Supra­molecular coordination geometry of the title compound in the crystal and (*b*) the corresponding polyhedron. Symmetry codes: (i) −1 + *x*, *y*, *z*; (ii) *x*, −1 + *y*, *z*; (iii) 1 + *x*, −1 + *y*, *z*; (iv) *x*, *y*, 1 + *z*. H atoms and isopropyl groups are omitted for clarity.

The mol­ecules of the title compound are well separated from each other in the crystal structure, with one magnesocene mol­ecule per unit cell. Each mol­ecule has eight neighboring mol­ecules, forming a distorted hexa­gonal bipyramidal coordination geometry (Fig. 3 ▸ *a* and 3*b*), with distances of *d*_min_ (Mg1⋯Mg1^i^) = 8.7025 (4) Å, *d*_max_ (Mg1⋯Mg1^iii^) = 9.3031 (3) Å and *d*_axial_ (Mg1⋯Mg1^iv^) = 9.2033 (4) Å [symmetry codes: (i) −1 + *x*, *y*, *z;* (iii) 1 + *x*, −1 + *y*, *z*; (iv) *x*, *y*, 1 + *z*]. The angles between the equatorial Mg atoms, the central magnesium atom and the axial magnesium atom are between θ_min_ = 90.68° (Mg1^iii^—Mg1—Mg1^iv^) and θ_max_ = 99.17° (Mg1^ii^—Mg1—Mg1^iv^).

**Figure 4:**
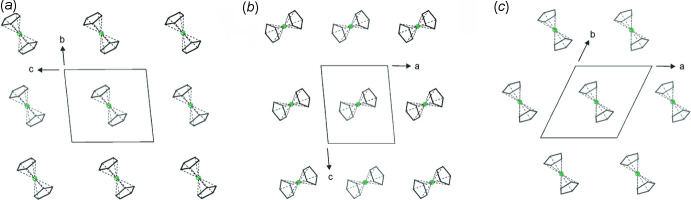
Arrangement of the layers of the title compound along the crystallographic *a*, *b* and *c* axes (H atoms and isopropyl groups omitted for clarity).

Each ^3^Cp_2_Mg moiety has eight neighboring mol­ecules within the *bc* and *ac* planes (Fig. 4 ▸ *a* and 4*b*), but only six neighboring mol­ecules within the *ab* plane, forming an almost hexa­gonal layer (γ = 63.00°), but with the layers being congruent to each other (Fig. 4 ▸ *c*).

## Database survey

A search in the Cambridge Structural Database (*CSD*, Version 5.42, update of September 2021; Groom *et al.*, 2016 ▸) showed that 14 crystal structures of magnesocenes of the type (C_5_ *R*_5_)_2_Mg had previously been reported. In this search, any type of donor complexes of magnesocenes of the form (C_5_ *R*_5_)_2_Mg**·** *D_n_* are not counted. The Mg⋯Cp^centroid^ bonding distances in these structures lie between 1.9562 (1) and 2.0628 (11) Å and the dihedral angles between the Cp planes are between 0° (co-planar geometry) and 17.892°. Thus, the bond distances and angles in the title compound are within normal ranges of known magnesocenes.

## Synthesis and crystallization

**Figure 5:**
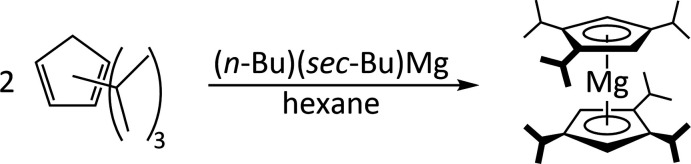
Reaction scheme for the formation of the title compound ^3^Cp_2_Mg.

Hanusa and coworkers had previously reported that 1,1′,2,2′,4,4′-hexa­iso­propyl­magnesocene, ^3^Cp_2_Mg, could be obtained by the reaction of potassium 1,2,4-triiso­propyl­cyclo­penta­dienide with magnesium(II) bromide. However, in this work, we utilized di­butyl­magnesium as a strong base to deprotonate the triiso­propyl­cyclo­penta­diene (Fig. 5 ▸).

To a solution of 4.00 g (20.8 mmol) of an isomeric mixture of triiso­propyl­cyclo­penta­diene in 100 mL of hexane were added 15.0 mL of a 0.7 *M* solution of *n*-butyl-*sec*-butyl­magnesium in hexane (10.5 mmol). The light-yellow reaction solution was stirred at 333 K overnight. Subsequently, all volatiles were removed *in vacuo* and a yellow oil was obtained, from which the title compound crystallized over the course of one day at ambient temperature. The crystallized material was washed with small portions of hexane and dried *in vacuo* to obtain the title compound as a pure, colorless, crystalline solid in 43% yield (1.83 g; 4.50 mmol).

In addition to a structural characterization by single-crystal X-ray diffraction, the title compound was also characterized by ^1^H and ^13^C NMR spectroscopy: ^1^H NMR (400 MHz, C_6_D_6_, 295 K): δ (in ppm) = 1.07 [*d*, *J* = 7Hz, 12H, CH(CH_3_)_2_], 1.28 [*d*, *J* = 7Hz, 12H, CH(CH_3_)_2_], 1.36 [*d*, *J* = 7Hz, 12H, CH(CH_3_)_2_], 2.82–2.92 [*m*, 6H, CH(CH_3_)_2_], 5.77 (*s*, 4H, Cp-H); ^1^H NMR (400 MHz, DMSO-D_6_, 294 K): δ (in ppm) = 1.06 [*d*, *J* = 7Hz, 36H, CH(CH_3_)_2_], 2.68 [*sep*, *J* = 7Hz, 2H, CH(CH_3_)_2_], 2.76 [*sep*, *J* = 7Hz, 2H, CH(CH_3_)_2_], 4.94 (*s*, 4H, Cp-H); ^13^C{^1^H} NMR (101 MHz, C_6_D_6_, 295 K): δ (in ppm) = 24.0 (^*i*^Pr), 24.4 (^*i*^Pr), 26.4 (^*i*^Pr), 26.6 (^*i*^Pr), 28.7 (^*i*^Pr), 98.7 (Cp), 125.3 (Cp), 128.6 (Cp); ^13^C{^1^H} NMR (101 MHz, DMSO-D_6_, 294 K): δ (in ppm) = 25.9 (^*i*^Pr), 26.8 (^*i*^Pr), 27.0 (^*i*^Pr), 29.1 (^*i*^Pr), 94.6 (Cp), 119.4 (Cp), 120.9 (Cp).

## Refinement

Crystal data, data collection and structure refinement details are summarized in Table 1 ▸. All non H-atoms were located in the electron density maps and refined anisotropically. C-bound H atoms were placed in positions of optimized geometry and treated as riding atoms: C—H = 1.00 Å (CH), 0.98 Å (CH_3_), and with *U*_iso_(H) = *kU*_eq_(C), where *k* = 1.2 for CH and 1.5 for CH_3_.

## Supplementary Material

Crystal structure: contains datablock(s) I. DOI: 10.1107/S2056989022001189/pk2661sup1.cif

Structure factors: contains datablock(s) I. DOI: 10.1107/S2056989022001189/pk2661Isup2.hkl

CCDC reference: 2149608

Additional supporting information:  crystallographic information; 3D view; checkCIF report

## Figures and Tables

**Table 1:** Experimental details

Crystal data	
Chemical formula	[Mg(C_14_H_23_)_2_]
*M*_r_	406.96
Crystal system, space group	Triclinic, *P* 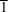
Temperature (K)	133
*a*, *b*, *c* (Å)	8.7025 (4), 9.0903 (4), 9.2033 (4)
α, β, γ (°)	80.829 (2), 81.151 (2), 63.004 (1)
*V* (Å^3^)	637.68 (5)
*Z*	1
Radiation type	Mo *K*α
μ (mm^−1^)	0.08
Crystal size (mm)	0.27 × 0.20 × 0.07

Data collection	
Diffractometer	Bruker D8 Venture Photon II
Absorption correction	Multi-scan (*SADABS*; Krause *et al.*, 2015 ▸)
*T*_min_, *T*_max_	0.712, 0.746
No. of measured, independent and observed [*I* > 2σ(*I*)] reflections	24343, 2808, 2339
*R*_int_	0.046
(sin θ/λ)_max_ (Å^−1^)	0.642

Refinement	
*R*[*F* ^2^ > 2σ(*F* ^2^)], *wR*(*F* ^2^), *S*	0.041, 0.100, 1.06
No. of reflections	2808
No. of parameters	138
H-atom treatment	H-atom parameters constrained
Δρ_max_, Δρ_min_ (e Å^−3^)	0.23, −0.20

Computer programs: *APEX3* and *SAINT* (Bruker, 2019 ▸), *SHELXT2018/2* (Sheldrick, 2015*a* ▸), *SHELXL2018/3* (Sheldrick, 2015*b* ▸), *shelXle* (Hübschle *et al.*, 2011 ▸) and *publCIF* (Westrip, 2010 ▸).

## supplementary crystallographic information

### Crystal data

**Table:**

[Mg(C_14_H_23_)_2_]	*Z* = 1
*M**_r_* = 406.96	*F*(000) = 226
Triclinic, *P*1	*D*_x_ = 1.060 Mg m^−^^3^
*a* = 8.7025 (4) Å	Mo *K*α radiation, λ = 0.71073 Å
*b* = 9.0903 (4) Å	Cell parameters from 7985 reflections
*c* = 9.2033 (4) Å	θ = 2.5–27.1°
α = 80.829 (2)°	µ = 0.08 mm^−^^1^
β = 81.151 (2)°	*T* = 133 K
γ = 63.004 (1)°	Plate, yellow
*V* = 637.68 (5) Å^3^	0.27 × 0.20 × 0.07 mm

### Data collection

**Table:**

Bruker D8 Venture Photon II diffractometer	2339 reflections with *I* > 2σ(*I*)
Radiation source: INCOATEC IÂµS microfocus sealed tube	*R*_int_ = 0.046
φ and ω scans	θ_max_ = 27.1°, θ_min_ = 2.3°
Absorption correction: multi-scan (SADABS; Krause *et al.*, 2015)	*h* = −11→11
*T*_min_ = 0.712, *T*_max_ = 0.746	*k* = −11→11
24343 measured reflections	*l* = −11→11
2808 independent reflections	

### Refinement

**Table:**

Refinement on *F*^2^	Primary atom site location: structure-invariant direct methods
Least-squares matrix: full	Secondary atom site location: difference Fourier map
*R*[*F*^2^ > 2σ(*F*^2^)] = 0.041	Hydrogen site location: inferred from neighbouring sites
*wR*(*F*^2^) = 0.100	H-atom parameters constrained
*S* = 1.06	*w* = 1/[σ^2^(*F*_o_^2^) + (0.0357*P*)^2^ + 0.2575*P*] where *P* = (*F*_o_^2^ + 2*F*_c_^2^)/3
2808 reflections	(Δ/σ)_max_ < 0.001
138 parameters	Δρ_max_ = 0.23 e Å^−^^3^
0 restraints	Δρ_min_ = −0.20 e Å^−^^3^

### Special details

**Table:**

Geometry. All esds (except the esd in the dihedral angle between two l.s. planes) are estimated using the full covariance matrix. The cell esds are taken into account individually in the estimation of esds in distances, angles and torsion angles; correlations between esds in cell parameters are only used when they are defined by crystal symmetry. An approximate (isotropic) treatment of cell esds is used for estimating esds involving l.s. planes.

### Fractional atomic coordinates and isotropic or equivalent isotropic displacement parameters (Å^2^)

**Table:**

	*x*	*y*	*z*	*U*_iso_*/*U*_eq_	
Mg1	0.500000	0.500000	0.500000	0.02198 (16)	
C1	0.58902 (16)	0.29370 (15)	0.34104 (14)	0.0220 (3)	
C2	0.68748 (15)	0.38509 (15)	0.29476 (14)	0.0222 (3)	
C3	0.78805 (16)	0.36201 (16)	0.41245 (14)	0.0230 (3)	
H3	0.876899	0.404101	0.408093	0.028*	
C4	0.75424 (15)	0.25725 (15)	0.53102 (14)	0.0223 (3)	
C5	0.63013 (16)	0.21621 (15)	0.48667 (14)	0.0232 (3)	
H5	0.587559	0.136717	0.544119	0.028*	
C6	0.47318 (17)	0.27043 (16)	0.24895 (15)	0.0257 (3)	
H6	0.444736	0.359348	0.164188	0.031*	
C7	0.30301 (19)	0.2858 (2)	0.33539 (19)	0.0371 (4)	
H7A	0.233030	0.269800	0.271125	0.056*	
H7B	0.327426	0.201156	0.420434	0.056*	
H7C	0.239326	0.396273	0.370110	0.056*	
C8	0.5672 (2)	0.10251 (19)	0.18531 (18)	0.0368 (4)	
H8A	0.491531	0.091596	0.123487	0.055*	
H8B	0.597174	0.012997	0.266371	0.055*	
H8C	0.673219	0.095503	0.125346	0.055*	
C9	0.69613 (17)	0.47897 (17)	0.14521 (15)	0.0261 (3)	
H9	0.585123	0.513065	0.101394	0.031*	
C10	0.7151 (2)	0.6358 (2)	0.15456 (18)	0.0422 (4)	
H10A	0.719212	0.692389	0.055076	0.063*	
H10B	0.822300	0.606089	0.198629	0.063*	
H10C	0.615986	0.709829	0.215971	0.063*	
C11	0.8434 (2)	0.3681 (2)	0.04173 (17)	0.0401 (4)	
H11A	0.843621	0.430001	−0.055715	0.060*	
H11B	0.827146	0.270336	0.032239	0.060*	
H11C	0.954060	0.332769	0.082140	0.060*	
C12	0.82800 (17)	0.20815 (17)	0.68044 (15)	0.0274 (3)	
H12	0.750729	0.296459	0.745989	0.033*	
C13	1.00801 (19)	0.1994 (2)	0.66748 (18)	0.0408 (4)	
H13A	1.087252	0.112649	0.604724	0.061*	
H13B	1.049205	0.173474	0.765945	0.061*	
H13C	1.003708	0.306602	0.623400	0.061*	
C14	0.8308 (3)	0.0458 (2)	0.75386 (19)	0.0463 (4)	
H14A	0.712755	0.055338	0.767566	0.069*	
H14B	0.876845	0.019984	0.850239	0.069*	
H14C	0.904440	−0.043197	0.691324	0.069*	

### Atomic displacement parameters (Å^2^)

**Table:**

	*U*^11^	*U*^22^	*U*^33^	*U*^12^	*U*^13^	*U*^23^
Mg1	0.0202 (3)	0.0207 (3)	0.0236 (3)	−0.0070 (2)	0.0002 (2)	−0.0067 (2)
C1	0.0213 (6)	0.0189 (6)	0.0243 (6)	−0.0064 (5)	−0.0012 (5)	−0.0066 (5)
C2	0.0195 (6)	0.0222 (6)	0.0227 (6)	−0.0066 (5)	0.0004 (5)	−0.0063 (5)
C3	0.0183 (6)	0.0259 (6)	0.0246 (6)	−0.0088 (5)	−0.0004 (5)	−0.0064 (5)
C4	0.0191 (6)	0.0209 (6)	0.0238 (6)	−0.0049 (5)	−0.0011 (5)	−0.0065 (5)
C5	0.0247 (6)	0.0196 (6)	0.0247 (6)	−0.0089 (5)	−0.0015 (5)	−0.0040 (5)
C6	0.0281 (7)	0.0234 (6)	0.0278 (7)	−0.0116 (5)	−0.0063 (5)	−0.0040 (5)
C7	0.0287 (7)	0.0408 (8)	0.0478 (9)	−0.0168 (6)	−0.0016 (6)	−0.0174 (7)
C8	0.0358 (8)	0.0375 (8)	0.0415 (9)	−0.0151 (7)	−0.0044 (7)	−0.0184 (7)
C9	0.0246 (6)	0.0300 (7)	0.0236 (7)	−0.0123 (5)	−0.0010 (5)	−0.0027 (5)
C10	0.0609 (11)	0.0354 (8)	0.0335 (8)	−0.0274 (8)	0.0068 (7)	−0.0033 (7)
C11	0.0467 (9)	0.0395 (8)	0.0274 (8)	−0.0156 (7)	0.0090 (7)	−0.0075 (6)
C12	0.0268 (7)	0.0270 (6)	0.0246 (7)	−0.0071 (5)	−0.0034 (5)	−0.0059 (5)
C13	0.0298 (8)	0.0566 (10)	0.0338 (8)	−0.0146 (7)	−0.0090 (6)	−0.0067 (7)
C14	0.0652 (11)	0.0427 (9)	0.0356 (9)	−0.0270 (9)	−0.0204 (8)	0.0085 (7)

### Geometric parameters (Å, º)

**Table:**

Mg1—C3^i^	2.3136 (12)	C7—H7B	0.9800
Mg1—C3	2.3136 (12)	C7—H7C	0.9800
Mg1—C5	2.3148 (12)	C8—H8A	0.9800
Mg1—C5^i^	2.3148 (12)	C8—H8B	0.9800
Mg1—C4	2.3253 (12)	C8—H8C	0.9800
Mg1—C4^i^	2.3253 (12)	C9—C11	1.5239 (19)
Mg1—C2^i^	2.3355 (12)	C9—C10	1.525 (2)
Mg1—C2	2.3355 (12)	C9—H9	1.0000
Mg1—C1^i^	2.3375 (12)	C10—H10A	0.9800
Mg1—C1	2.3376 (12)	C10—H10B	0.9800
C1—C2	1.4237 (18)	C10—H10C	0.9800
C1—C5	1.4277 (18)	C11—H11A	0.9800
C1—C6	1.5143 (17)	C11—H11B	0.9800
C2—C3	1.4268 (17)	C11—H11C	0.9800
C2—C9	1.5101 (18)	C12—C14	1.514 (2)
C3—C4	1.4172 (19)	C12—C13	1.519 (2)
C3—H3	1.0000	C12—H12	1.0000
C4—C5	1.4220 (18)	C13—H13A	0.9800
C4—C12	1.5226 (18)	C13—H13B	0.9800
C5—H5	1.0000	C13—H13C	0.9800
C6—C7	1.527 (2)	C14—H14A	0.9800
C6—C8	1.5317 (18)	C14—H14B	0.9800
C6—H6	1.0000	C14—H14C	0.9800
C7—H7A	0.9800		
			
C3^i^—Mg1—C3	180.0	C5—C4—C12	126.88 (12)
C3^i^—Mg1—C5	121.04 (5)	C3—C4—Mg1	71.76 (7)
C3—Mg1—C5	58.96 (5)	C5—C4—Mg1	71.75 (7)
C3^i^—Mg1—C5^i^	58.96 (5)	C12—C4—Mg1	118.67 (8)
C3—Mg1—C5^i^	121.04 (5)	C4—C5—C1	109.12 (11)
C5—Mg1—C5^i^	180.0	C4—C5—Mg1	72.56 (7)
C3^i^—Mg1—C4	144.42 (5)	C1—C5—Mg1	73.00 (7)
C3—Mg1—C4	35.58 (5)	C4—C5—H5	125.3
C5—Mg1—C4	35.69 (4)	C1—C5—H5	125.3
C5^i^—Mg1—C4	144.31 (4)	Mg1—C5—H5	125.3
C3^i^—Mg1—C4^i^	35.58 (5)	C1—C6—C7	112.68 (11)
C3—Mg1—C4^i^	144.42 (5)	C1—C6—C8	110.81 (11)
C5—Mg1—C4^i^	144.31 (4)	C7—C6—C8	109.65 (12)
C5^i^—Mg1—C4^i^	35.69 (4)	C1—C6—H6	107.8
C4—Mg1—C4^i^	180.0	C7—C6—H6	107.8
C3^i^—Mg1—C2^i^	35.74 (4)	C8—C6—H6	107.8
C3—Mg1—C2^i^	144.26 (4)	C6—C7—H7A	109.5
C5—Mg1—C2^i^	120.74 (5)	C6—C7—H7B	109.5
C5^i^—Mg1—C2^i^	59.26 (5)	H7A—C7—H7B	109.5
C4—Mg1—C2^i^	120.27 (4)	C6—C7—H7C	109.5
C4^i^—Mg1—C2^i^	59.73 (4)	H7A—C7—H7C	109.5
C3^i^—Mg1—C2	144.26 (4)	H7B—C7—H7C	109.5
C3—Mg1—C2	35.74 (4)	C6—C8—H8A	109.5
C5—Mg1—C2	59.26 (5)	C6—C8—H8B	109.5
C5^i^—Mg1—C2	120.74 (5)	H8A—C8—H8B	109.5
C4—Mg1—C2	59.73 (4)	C6—C8—H8C	109.5
C4^i^—Mg1—C2	120.27 (4)	H8A—C8—H8C	109.5
C2^i^—Mg1—C2	180.0	H8B—C8—H8C	109.5
C3^i^—Mg1—C1^i^	59.17 (4)	C2—C9—C11	110.83 (11)
C3—Mg1—C1^i^	120.83 (4)	C2—C9—C10	112.69 (11)
C5—Mg1—C1^i^	144.26 (4)	C11—C9—C10	109.89 (12)
C5^i^—Mg1—C1^i^	35.74 (4)	C2—C9—H9	107.7
C4—Mg1—C1^i^	120.28 (4)	C11—C9—H9	107.7
C4^i^—Mg1—C1^i^	59.72 (4)	C10—C9—H9	107.7
C2^i^—Mg1—C1^i^	35.48 (4)	C9—C10—H10A	109.5
C2—Mg1—C1^i^	144.52 (4)	C9—C10—H10B	109.5
C3^i^—Mg1—C1	120.83 (4)	H10A—C10—H10B	109.5
C3—Mg1—C1	59.17 (4)	C9—C10—H10C	109.5
C5—Mg1—C1	35.74 (4)	H10A—C10—H10C	109.5
C5^i^—Mg1—C1	144.26 (4)	H10B—C10—H10C	109.5
C4—Mg1—C1	59.72 (4)	C9—C11—H11A	109.5
C4^i^—Mg1—C1	120.28 (4)	C9—C11—H11B	109.5
C2^i^—Mg1—C1	144.52 (4)	H11A—C11—H11B	109.5
C2—Mg1—C1	35.48 (4)	C9—C11—H11C	109.5
C1^i^—Mg1—C1	180.0	H11A—C11—H11C	109.5
C2—C1—C5	107.47 (11)	H11B—C11—H11C	109.5
C2—C1—C6	126.57 (12)	C14—C12—C13	110.23 (13)
C5—C1—C6	125.78 (12)	C14—C12—C4	112.14 (12)
C2—C1—Mg1	72.18 (7)	C13—C12—C4	111.48 (12)
C5—C1—Mg1	71.26 (7)	C14—C12—H12	107.6
C6—C1—Mg1	125.75 (8)	C13—C12—H12	107.6
C1—C2—C3	107.35 (11)	C4—C12—H12	107.6
C1—C2—C9	126.99 (11)	C12—C13—H13A	109.5
C3—C2—C9	125.51 (12)	C12—C13—H13B	109.5
C1—C2—Mg1	72.34 (7)	H13A—C13—H13B	109.5
C3—C2—Mg1	71.29 (7)	C12—C13—H13C	109.5
C9—C2—Mg1	125.19 (8)	H13A—C13—H13C	109.5
C4—C3—C2	109.38 (11)	H13B—C13—H13C	109.5
C4—C3—Mg1	72.66 (7)	C12—C14—H14A	109.5
C2—C3—Mg1	72.97 (7)	C12—C14—H14B	109.5
C4—C3—H3	125.2	H14A—C14—H14B	109.5
C2—C3—H3	125.2	C12—C14—H14C	109.5
Mg1—C3—H3	125.2	H14A—C14—H14C	109.5
C3—C4—C5	106.69 (11)	H14B—C14—H14C	109.5
C3—C4—C12	126.32 (12)		
			
C5—C1—C2—C3	0.18 (13)	C2—C1—C5—C4	−0.49 (13)
C6—C1—C2—C3	−175.17 (11)	C6—C1—C5—C4	174.90 (11)
Mg1—C1—C2—C3	63.12 (8)	Mg1—C1—C5—C4	−64.04 (8)
C5—C1—C2—C9	175.90 (11)	C2—C1—C5—Mg1	63.55 (8)
C6—C1—C2—C9	0.6 (2)	C6—C1—C5—Mg1	−121.06 (12)
Mg1—C1—C2—C9	−121.16 (12)	C2—C1—C6—C7	−138.10 (13)
C5—C1—C2—Mg1	−62.94 (8)	C5—C1—C6—C7	47.38 (17)
C6—C1—C2—Mg1	121.71 (12)	Mg1—C1—C6—C7	−44.39 (16)
C1—C2—C3—C4	0.21 (14)	C2—C1—C6—C8	98.62 (15)
C9—C2—C3—C4	−175.60 (11)	C5—C1—C6—C8	−75.90 (16)
Mg1—C2—C3—C4	64.02 (9)	Mg1—C1—C6—C8	−167.68 (10)
C1—C2—C3—Mg1	−63.81 (8)	C1—C2—C9—C11	−90.94 (15)
C9—C2—C3—Mg1	120.39 (12)	C3—C2—C9—C11	84.04 (16)
C2—C3—C4—C5	−0.50 (13)	Mg1—C2—C9—C11	175.25 (10)
Mg1—C3—C4—C5	63.71 (8)	C1—C2—C9—C10	145.44 (13)
C2—C3—C4—C12	−176.84 (11)	C3—C2—C9—C10	−39.58 (18)
Mg1—C3—C4—C12	−112.63 (12)	Mg1—C2—C9—C10	51.63 (15)
C2—C3—C4—Mg1	−64.21 (8)	C3—C4—C12—C14	−155.61 (13)
C3—C4—C5—C1	0.61 (13)	C5—C4—C12—C14	28.78 (18)
C12—C4—C5—C1	176.92 (11)	Mg1—C4—C12—C14	116.73 (12)
Mg1—C4—C5—C1	64.33 (8)	C3—C4—C12—C13	−31.46 (18)
C3—C4—C5—Mg1	−63.71 (8)	C5—C4—C12—C13	152.93 (13)
C12—C4—C5—Mg1	112.60 (12)	Mg1—C4—C12—C13	−119.13 (11)

Symmetry code: (i) −*x*+1, −*y*+1, −*z*+1.
